# A polarization-independent blue phase liquid crystal on silicon with low operation voltage

**DOI:** 10.1038/s41598-019-53344-6

**Published:** 2019-11-15

**Authors:** Changli Sun, Jiangang Lu

**Affiliations:** Department of Electronic Engineering, Shanghai Jiao Tong University, Shanghai, 200240 China

**Keywords:** Liquid crystals, Silicon photonics

## Abstract

A polarization-independent blue phase liquid crystal on silicon (BPLCoS) device with low operation voltage for 2π phase depth is demonstrated. With optimized reflection structure and two reflection films, the incident light may experience multifold optical path and 2π phase depth can be obtained. For the polarization-independence, an inclined electric field made by periodical gradient voltage is applied on the blue phase liquid crystal (BPLC) to match the light propagation direction. With the structure, the operation voltage can be lowered to 5.5 V in simulation and 5.9 V in experiment for 2π phase modulation at 1550 nm. The proposed device shows great potential for communication and imaging systems.

## Introduction

A liquid crystal spatial light modulator (SLM) is important in many applications, such as adaptive optics^1^, laser beam steering^2^, and optical communications^3^. For a liquid crystal on silicon (LCoS) device, polarization independence, high phase depth and high driving capacity are highly desirable^4–8^. Several types of LCs have been used in the device to realize polarization independence, such as polymer network liquid crystal^9–12^, polymer-dispersed liquid crystal^13–15^, and blue phase liquid crystal^16–18^. Among them, polymer-stabilized blue phase liquid crystal (PS-BPLC) based LCoS is particularly promising because of its macro optical isotropic status, simple fabrication process and sub-millisecond response time^16,19^. Rachel M. Hyman *et al*. demonstrated a polarization-independent PS-BPLC SLM with 1.8π phase depth at an applied field of 20 V·μm^−1^ at 652 nm^20^. However, some issues limit the wide application of PS-BPLC, especially the high driving voltage for 2π phase depth. Several methods have been proposed to improve the electro-optic property of blue phase liquid crystal on silicon (BPLCoS) devices. Fenglin Peng *et al*. proposed a new polarization-independent blue phase liquid crystal on silicon device with 2π phase depth below 24 V in the visible region by using a reflective polarizer which allowed the incident light beam to traverse four times inside the BPLC layer^21^. The 2π phase depth voltage of the BPLCoS device was lowered to 26 V by optimizing the material parameters and cell gap thickness^16^. However, the operation voltage for 2π phase depth of the device is still relatively high, which is not low enough to be used on silicon.

In this work, a polarization-independent BPLCoS device with low operation voltage for 2π phase depth is proposed. With optimized reflection structure and two reflection films, the incident light may experience multifold optical path and 2π phase depth can be obtained. For the polarization-independence, an inclined electric field made by periodical gradient voltage is applied on the blue phase liquid crystal to match the light propagation direction. The proposed device shows great potential for communication and imaging systems.

## Design Principle

**Figure 1:**
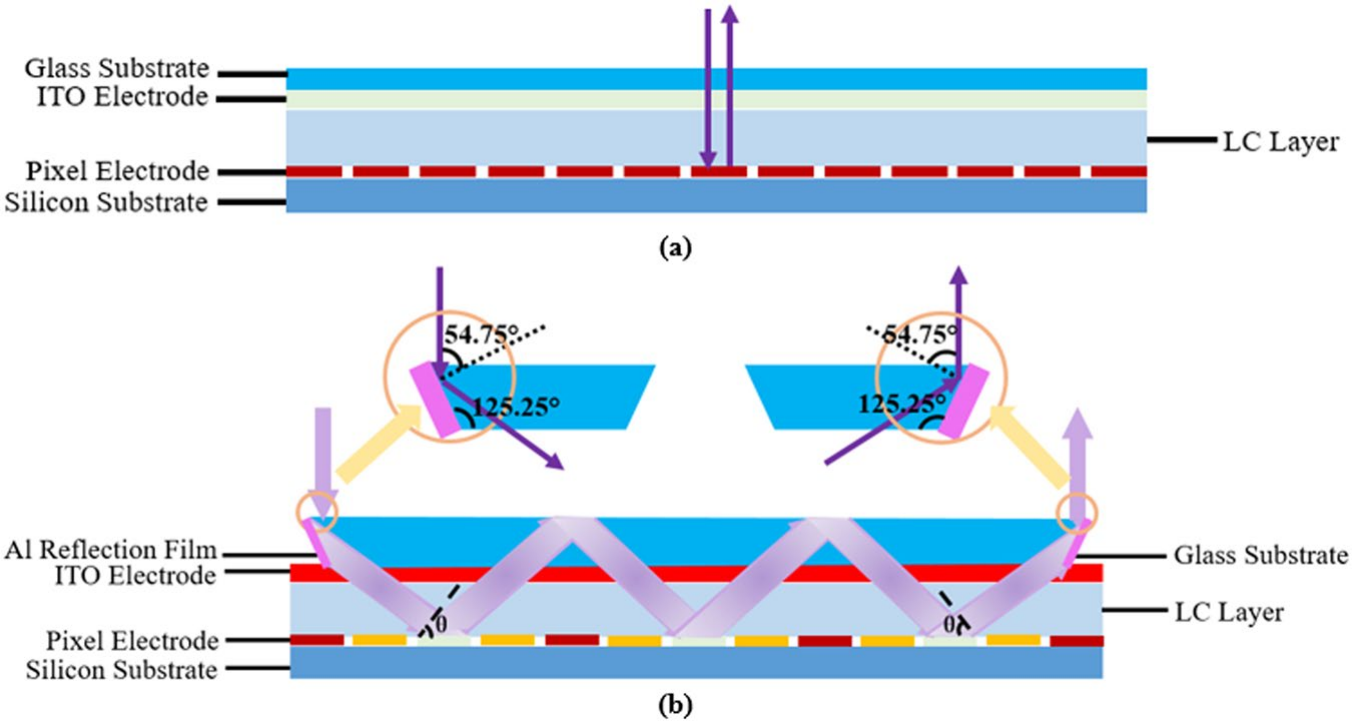
Schematic diagrams of (**a**) a normal LCoS structure and (**b**) the proposed LCoS structure.

Figure 1 shows the schematic diagrams of a normal LCoS structure (Fig. 1(a)) and the proposed LCoS structure. In the normal LCoS structure, the LC layer is sandwiched between the glass substrate covered with indium-tin-oxide (ITO) electrode and silicon substrate covered with Al pixel electrode. In this structure, the light beam only traverses the LC layer twice, leading to a low phase depth and a high 2π phase depth operation voltage. Figure 1(b) depicts the device configuration and operation mechanism of the proposed polarization-independent BPLCoS device. The BPLC is sandwiched between pixel electrodes and the common indium-tin-oxide electrode. Because the refractive index of the air is lower than that of the glass substrate (n = 1.5), the light beam will be reflected by the glass substrate if the incident angle on the interface is larger than the critical angle for the total internal reflection. For a given BPLC layer thickness, a large incident angle can lead to a large phase depth and a low operation voltage. Therefore, an Al reflection film is embedded in the left edge of the glass substrate to redirect the normally incident light. In our design, the divergence angle of the light beam is 0 mrad ideally. The widest diameter of the beam can be obtained by multiplying the pixel size by cosθ. For a pixel size of 6.4 um and θ of 70.5°, the beam diameter is calculated as 2.14 um. In our design, the light beam in micron size can be used as the light source^22^. The oblique incident angle, θ, should be larger than the critical angle to ensure the total internal reflection on the interface between the air and the glass substrate. Based on the law of the total internal reflection, the critical angle on the interface between the air and the glass substrate is calculated as 42°. In order to prolong the optical path length and realize the total internal reflection, the incident angle θ in the BPLC layer is designed as 70.5°. According to θ and the law of reflection, as well as to obtain the normally input and output light, the internal angles of the top glass substrate are designed at 54.75°–125.25°–125.25°–54.75°. Different driving voltages are applied on the discrete pixel electrodes to form periodical and symmetric gradient electric fields, matching the light propagation direction. The output light beam reflected by the pixel electrode is redirected by the right Al reflection film, the location of which determines the optical path length in the BPLC layer. In our design, the light beam traverses obliquely six times inside the BPLC layer before exiting the LCoS device. With the cell gap of 6 um, the optical path length in our proposed structure is seven times of the normal LCoS structure.

Because it’s difficult to obtain a laser with diameter of less than 2.14 um, in experiment we use an equivalent laser. The divergence angle and diameter of the laser are 2.5 mrad and 1 mm, respectively. Due to the complex fabrication process of the device structure, a device with equivalent structure of several mirrors to multiply the optical path is designed for the measurement. As the gradient electric field direction is approximately parallel to the light propagation direction in simulation, a cell comprised of two ITO glass substrates is applied to measure the phase depth. Because there is a little deviation between the gradient electric field direction and the light propagation direction in simulation, the cell is set with an inclination angle to measure the polarization independence.

## Simulation

**Figure 2:**
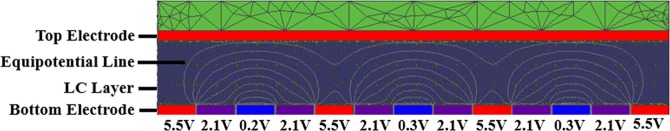
The electric field equipotential line distribution of the device in simulation.

The electric field distribution of the proposed device is simulated by the software DIMOS.2d. The equipotential lines (white lines), the electrode voltage distribution, and the structure of the device in simulation are shown Fig. 2. To form a gradient electric field, the optimized driving voltages applied on pixel electrodes in one period are 5.5 V, 2.1 V, 0.2 V, 2.1 V, and 5.5 V. The driving voltage applied on the ITO electrode is set as 5.5 V. The pixel size and the pixel gap are 6.4 um and 0.2 um, respectively, and the BPLC layer thickness is 6 um. The LC material LC-S1 (Δn = 0.28, Δε = 15.6, γ = 0.149 pa·s, at λ = 1550 nm) is employed as the nematic liquid crystal host of the blue phase module in the simulation. The Kerr constant of the PS-BPLC material (BP I) is 10.7 nm·V^−2^ at 1550 nm. The induced birefringence under the electric field can be obtained on the basis of Kerr effect, and then the phase depth can be calculated. For the proposed structure, the phase depth difference between the phase depth with the applied voltage and that in the voltage-off state reaches 2π at 1550 nm. In the simulation with the structure in Fig. 1(a), the phase depth difference between the phase depth with 5.5 V on all the pixel electrodes and that in the voltage-off state is only 0.29π at 1550 nm.

**Figure 3:**
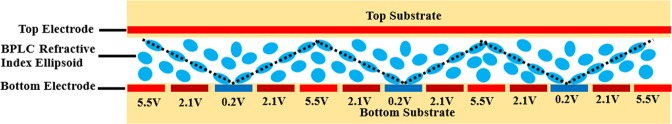
The diagram of the refractive index ellipsoid distribution of the BPLC material in simulation.

The diagram of the refractive index ellipsoid distribution of the BPLC material in simulation is shown in Fig. 3. The dashed lines represent the light and the blue ellipsoid is the refractive index ellipsoid of the BPLC material. The refractive index ellipsoid distribution in three periods is illustrated and the voltages on the electrodes are the same as those in simulation. The refractive index ellipsoid of the BPLC material can be elongated along the external electric field direction because of the Kerr effect^23^. And the BPLC material is polarization independent if the light transmits along the major axis of the refractive index ellipsoid^24^. In simulation, a gradient electric field is formed to make the major axis of the refractive index ellipsoid of the BPLC material arrange along the light propagation direction. Because the beam size is assumed in micron, it can transmit along the major axis of the refractive index ellipsoid, realizing the characteristic of polarization independence.

**Figure 4:**
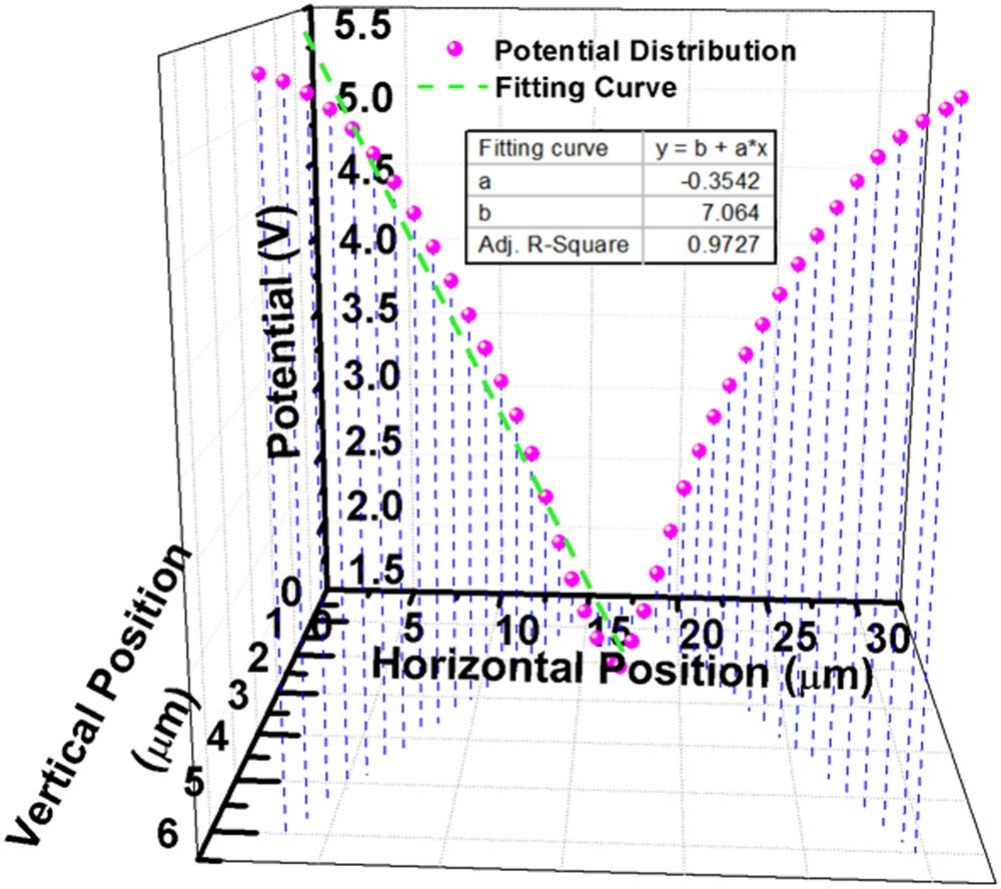
The electric potential distribution of the device in simulation.

Based on the simulation with the proposed structure, the electric potential distribution with horizontal position from 3.2 um to 29.6 um is calculated and the fitting line of the electric potential distribution is shown in Fig. 4. The parameter adjusted R-square of the fitting line describes the fitting degree, and a high value near 100% indicates that there is a good agreement between the experimental data and the fitting function. The parameter adjusted R-square, *R*^2^, can be described by Eq. (1):

1 $${R}^{2}=1-\frac{{\sum }_{i=1}^{n}{({y}_{i}-{Y}_{i})}^{2}}{{\sum }_{i=1}^{n}{({Y}_{i}-\bar{Y})}^{2}},$$

where *Y*_*i*_ and *y*_*i*_ represent the *i*th measured data value and the *i*th estimated data value, respectively, $\bar{Y}$ is the average value of the measured data, and *n* is the number of the data. According to the Eq. (1), the adjusted R-square of the fitting curve is calculated as 97.27%. Because the BPLC is optically isotropic only along the induced electric field direction, we consider that the polarization independence of the device is 97.27% if the light beam propagates along the direction of the fitting curve. The slope and intercept of the fitting curve are −0.3542 and 7.064, respectively. According to the slope and intercept of the fitting curve, the inclination angle of the fitting curve is calculated as 19.5°, corresponding to the incident angle, as well as the complementary angle, of 70.5°. Therefore, the optical path length in three periods can be calculated as 84 um, and is six times more than that in a normal LCoS structure.

## Experiments

### Measurement of phase depth

**Figure 5:**
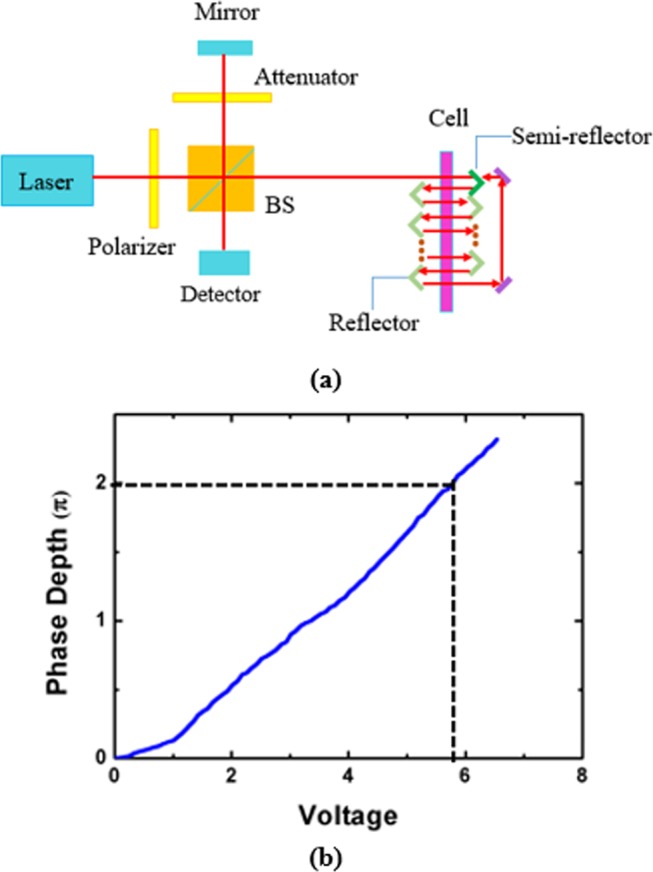
(**a**) Optical schematic diagram of the phase depth measurement system. (**b**) The phase depth versus voltage.

In order to investigate the electro-optic performance of the proposed BPLCoS device, we use the measurement system shown in Fig. 5(a) to realize the same optical path length of 84 um as the proposed structure because it’s difficult to obtain a laser with diameter of less than 2.14 um. The optical schematic diagram of the phase depth measurement system is shown in Fig. 5(a). The 1550 nm laser beam is divided into two halves by the beam splitter (BS) cube. One half transmits through the BS cube to the BPLC cell with cell gap of 6 um, while the other half is reflected to a flat mirror. In the cell, both the top and bottom glass substrates are over-coated with thin ITO electrodes. An attenuator is placed between the BS cube and the flat mirror to adjust the light intensity. When an electric field is applied on the top and bottom ITO electrodes of the cell, a phase difference will be formed between the beam back from the cell and the beam reflected by the mirror. Eleven rectangular mirrors, one semi-reflector, and two flat reflectors are placed around the cell to induce an 84 um optical path length inside the BPLC cell. The inner plane of the semi-reflector can reflect the light beam, while the outer plane will transmit the light beam. In order to guide the light beam, the inclination angle of the rectangular mirror is set as 45°, and the light will be reflected by each mirror twice. After reflected by the mirrors thirteen times, the light beam is reflected by a flat reflector to a second flat reflector, and then transmits through the semi-reflector. The light from the BPLC cell will be reflected by the BS cube, and then converges with the light from the mirror. The intensity of the interference beam is recorded by the power meter. When the voltage is applied on the top and bottom ITO electrodes of the cell, the refractive index ellipsoid of the BPLC material will be elongated along the external electric field direction because of the Kerr effect. In this state, the BPLC material is polarization independent, which is the same as that in simulation. The measured phase depth as a function of voltage is shown in Fig. 5(b), indicating that the 2π phase depth voltage is 5.9 V, which is in a good agreement with the simulation result. We have also measured the response time of the cell, which is 1.18 ms with the rise time of 320 us and fall time of 861 us.

### Measurement of polarization dependence

**Figure 6:**
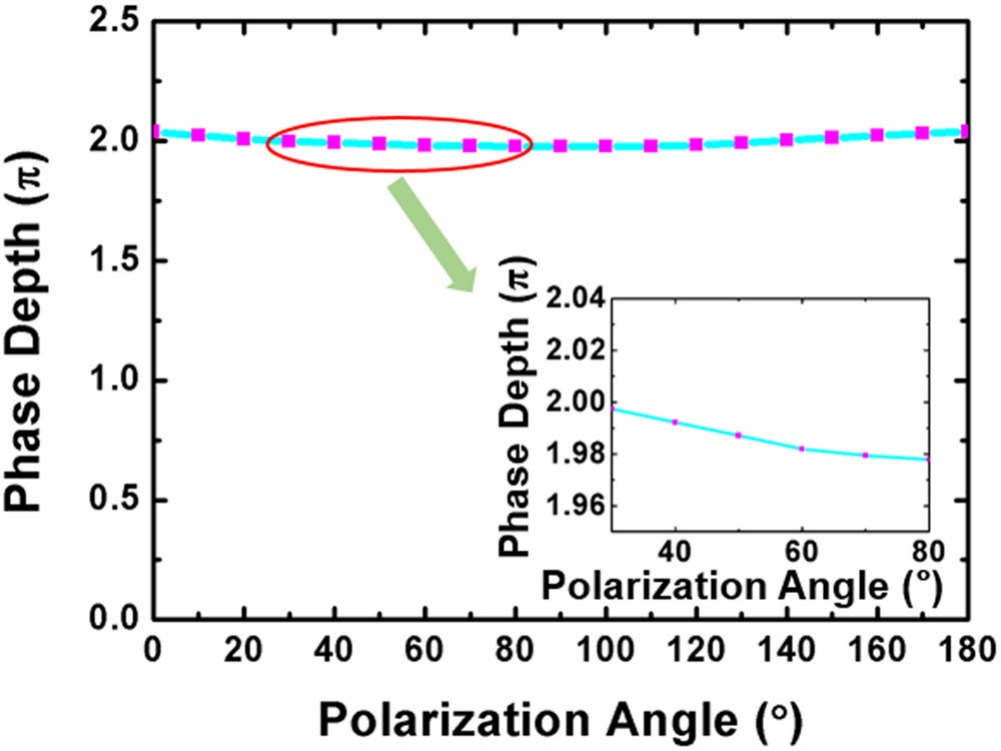
The polarization dependence of phase depth with inclination angle of 2.5°.

In simulation, the polarization independence is 97.27%, corresponding to the polarization dependence of 2.73%. In order to estimate the polarization dependence in experiment, we set the BPLC cell at an inclination angle of 2.5°, which is obtained by multiplying the normally incident angle of 90° by the polarization dependence of 2.73%. The polarization dependence measurement system is similar to the phase depth measurement system, except for the inclination angle of the cell. The polarization state of the light can be controlled by rotating the laser beam. The polarization state of the polarizer is adjusted to be the same as that of the laser beam. The BPLC cell with 6 um cell gap is set between rectangular mirrors at an inclination angle of 2.5°. The cell is applied with voltage from 0 to 5.9 V to realize a phase depth of 2π. When the polarization angles of polarizers are rotated from 0° to 180° at a step of 10°, the maximum phase depth values are recorded. The polarization dependence of phase depth with inclination angle of 2.5° is shown in Fig. 6, and the inset depicts the phase depth value with polarization angle from 30° to 80°. We assume that the value of the polarization dependence *P*_*D*_ can be calculated by the following equation:

2 $${P}_{D}=\frac{{P}_{max}-{P}_{min}}{{P}_{av}},$$

where *P*_max_ and *P*_min_ represent the maximum and the minimum phase depth value among the measured data, and *P*_av_ is the average of all the phase depth values. The difference between *P*_max_ and *P*_min_ represents the maximum fluctuation of the phase depth, and *P*_av_ represents the reference phase depth value. The polarization dependence *P*_*D*_ can describe the degree of the fluctuation in phase depth, and therefore it can describe the polarization dependence degree of the BPLC cell. The result in Fig. 6 shows that the phase depth fluctuates with the polarization angle only in a small range. By calculation with the Eq. (2), the value of the polarization dependence is calculated as only 3.09%, so that the device can be almost considered as polarization independent.

## Conclusions

A polarization-independent BPLCoS device with low operation voltage for 2π phase depth is demonstrated. With optimized reflection structure and two reflection films, the incident light may experience multifold optical path length and 2π phase depth can be obtained. For the polarization-independence, an inclined electric field made by periodical gradient voltage is applied on the blue phase liquid crystal to match the light propagation direction. Simulation and experiments have been done to measure the polarization dependence and phase depth. The 2π phase depth voltage can be lowered to 5.5 V in simulation and 5.9 V in experiment at 1550 nm. The proposed device shows great potential for communication and imaging systems.

## Data Availability

The datasets generated during the current study are available from the corresponding authors on reasonable request.
